# Use of the Zebrafish Larvae as a Model to Study Cigarette Smoke Condensate Toxicity

**DOI:** 10.1371/journal.pone.0115305

**Published:** 2014-12-19

**Authors:** Lee D. Ellis, Evelyn C. Soo, John C. Achenbach, Michael G. Morash, Kelly H. Soanes

**Affiliations:** 1 National Research Council of Canada, Aquatic and Crop Resource Development, 1411 Oxford Street, Halifax, Nova Scotia, B3H 3Z1, Canada; 2 Science Division, Office of Research and Surveillance, Controlled Substances and Tobacco Directorate, Health Canada, 150 Tunney's Pasture Driveway, Ottawa, Ontario, K1A 0K9, Canada, Locator: A.L. 0301A; Oregon State University, United States of America

## Abstract

The smoking of tobacco continues to be the leading cause of premature death worldwide and is linked to the development of a number of serious illnesses including heart disease, respiratory diseases, stroke and cancer. Currently, cell line based toxicity assays are typically used to gain information on the general toxicity of cigarettes and other tobacco products. However, they provide little information regarding the complex disease-related changes that have been linked to smoking. The ethical concerns and high cost associated with mammalian studies have limited their widespread use for *in vivo* toxicological studies of tobacco. The zebrafish has emerged as a low-cost, high-throughput, *in vivo* model in the study of toxicology. In this study, smoke condensates from 2 reference cigarettes and 6 Canadian brands of cigarettes with different design features were assessed for acute, developmental, cardiac, and behavioural toxicity (neurotoxicity) in zebrafish larvae. By making use of this multifaceted approach we have developed an *in vivo* model with which to compare the toxicity profiles of smoke condensates from cigarettes with different design features. This model system may provide insights into the development of smoking related disease and could provide a cost-effective, high-throughput platform for the future evaluation of tobacco products.

## Introduction

The link between tobacco use and the development of serious diseases such as certain cancers, heart disease, respiratory diseases and stroke are well established [1]. Despite this, tobacco use continues to be a growing global epidemic and remains the leading cause of preventable death worldwide [2]. More than 37,000 Canadians have been estimated to die prematurely on an annual basis as a consequence of tobacco smoking [3] and costs to the health care system are estimated at over $4.3 billion annually [4]. As such, tobacco use remains a public health concern in Canada.

Cigarettes are the most widely consumed tobacco product in Canada [5]. The majority of cigarettes sold in the Canadian market are similar in terms of their dimensions, the presence of a ventilated cellulose acetate filter, and in the exclusive use of Virginia flue-cured tobacco [6]. However, the tobacco industry has steadily introduced into the Canadian market cigarettes with other features such as the charcoal filter, as well as those that contain blends of different tobacco types. More recently, cigarettes featuring a novel copper phthalocyanine-containing filter [7], [8] or a super slim design [9] were launched in Canada, and information on the toxicity profiles of these new cigarettes is not widely available.

Under the Canadian *Tobacco Reporting Regulations* [10], tobacco product manufacturers and importers must provide Health Canada with information on the toxicity of mainstream smoke emissions of cigarettes sold in the Canadian market. Three cell-based assays are currently specified in the regulations to evaluate cigarette smoke condensates (CSCs) prepared from mainstream smoke for mutagenic, cytotoxic and clastogenic potential. While these tests are relatively high-throughput and low cost, they can only be used to assess general toxicity of tobacco products at the cellular level and are of limited use for understanding the consequences of exposure to cigarette smoke in an intact organism. These established *in vitro* assays lack the complex environment, interaction and influence of different cell types and tissues found in an *in vivo* system and therefore, due to their simplicity, provide a limited understanding of the potential harm associated with the CSCs. Unfortunately, the use of mammalian models for these purposes is time-consuming, prohibitively expensive and often raises ethical concerns. In contrast, it is becoming well known that the zebrafish toxicity model provides more detailed *in vivo* information than can be acquired from cell lines regarding developmental, cardiac and neurotoxicity [11]–[13]. Additionally, these studies can be performed for a fraction of the cost and in a shorter period of time than mammalian studies.

Zebrafish have been used for predicting vertebrate toxicity, teratogenicity and environmental risk assessment on a wide range of compounds focusing on morphological, molecular and preclinical safety pharmacological endpoints [14]–[17]. The zebrafish embryonic and larval stages are well suited for capturing information regarding not only the acute toxicity but also the developmental, cardiac and behavioural (neuro) toxicity of new chemical entities or complex mixtures.

While the focus of much of the research on tobacco has been on the toxicity profiles with respect to cancer and cardiovascular disease [18], the neurotoxic potential and effects on neuronal activity is important when considering the long-term cognitive effects of tobacco product use. Recent work has demonstrated the neurotoxic effects of nicotine on zebrafish [19]–[21] and a number of studies from the US Environmental Protection Agency have begun to standardize the assessment of changes in zebrafish larval behaviour, following exposure to toxins, as a means of assessing the neurotoxicity of a broad range of toxins [14], [15], [22]–[26]. While toxicity studies using the zebrafish also do not permit the evaluation of harm of tobacco products to human health, it is expected that they would likely yield more relevant toxicological information than *in vitro* and cell based assays.

As the utility of using zebrafish larvae as a model for studying tobacco product toxicity has not been evaluated, we have tested CSCs from two reference cigarettes and six commercial brands of Canadian cigarettes for acute, developmental, cardiac and behavioural toxicity (neurotoxicity) in zebrafish larvae, along with the underlying changes in gene expression associated with CSC exposure. By making use of this multifaceted approach, we were able to conduct a preliminary assessment of zebrafish embryos and larvae as a model for studying the potential adverse effects associated with CSC exposure and have generated toxicity profiles that may reflect potential harm that is relevant to other vertebrates.

## Materials and Methods

### Animals

Zebrafish (*Danio rerio)* were maintained according to standard animal care protocols [32] and in accordance with the Canadian Council on Animal Care guidelines. The research protocol was approved by the National Research Council of Canada Animal Care Committee in Halifax, Nova Scotia, protocol #2012-01. AB/Tubingen adults, embryos and larvae were maintained on a re-circulating Tecniplast aquatic system at 28°C±1°C and between pH 7.0–7.5 on a 14/10 h light/dark (L/D) cycle. Embryos were collected from multiple AB/Tubingen breeding pairs and pooled. Following 4–6 hours in an incubator in E3 media (5 mM NaCl, 0.17 mM KCl, 0.33 mM CaCl_2_•2H_2_O, 0.33 mM MgSO_4_) unfertilized embryos were removed. Larvae were placed in Aquatic Habitats mesh-bottom nursery baskets on the recirculation system until used. Following experimental procedures larvae were euthanized with a lethal dose of tricaine methanesulfonate.

### Cigarette selection

The Kentucky 3R4F reference cigarette (3R4F) and the Canadian Monitor 8 (CM8) were used as reference cigarettes for this study (**Table 1**). The 3R4F reference cigarette is an internationally recognized standard and typically used as a reference cigarette during smoke emission testing [27]. The 3R4F features a ventilated cellulose acetate filter and a blend of different tobacco types that is typical of the cigarettes sold in many countries including the United States [28]. The CM8 contains exclusively Virginia flue-cured tobacco, which is typical of Canadian commercial cigarettes, and a non-ventilated cellulose acetate filter. Both reference cigarettes have dimensions (circumference and lengths) of the majority of cigarettes sold in Canada. Six commercial brands of Canadian cigarettes were also selected for the study (**Table 1**) and these included a Canadian best seller (BSV), which has design features that were considered to be typical of the cigarettes sold in the Canadian market, and 5 other brands representing the different cigarette design features that were available in the Canadian market at the time of the study. Specifically, these included cigarettes containing a novel filter containing copper phthalocyanine (MBV), a blend of different tobacco types (MIX) or a super slim design (SSV, SSMIXL, SSC). Among the super slim cigarettes, one brand contained a blend of different tobacco types (SSMIX) and another brand contained a charcoal filter (SSC). With the exception of MIX and SSMIX, all the commercial cigarettes used in this study contained exclusively Virginia flue-cured tobacco.

**Table 1:** Characteristics of Cigarettes Selected for the Study.

Sample ID	Description	Length (mm)	Circumference (mm)	ISO^a^				HCI^b^			
				TPM^c^		Nicotine		TPM^c^		Nicotine	
				mg/cig	SD	mg/cig	SD	mg/cig	SD	mg/cig	SD
3R4F	3R4F Kentucky Reference	84	24±1.0	9.5	0.5	0.7	0.0	42.9	3.6	1.9	0.1
CM8	Canadian Monitor 8	84	24±1.0	16.0	0.9	1.1	0.0	44.7	2.6	1.9	0.1
BSV	Canadian Best Seller	72	24±1.0	13.6	1.1	0.8	0.0	47.7	2.9	1.9	0.1
MIX	Mixed blend^d^	72	24±1.0	17.3	1.1	1.1	0.1	50.3	2.4	2.4	0.1
MBV	Copper phthalocyanine containing Filter (MBV)	84	24±1.0	15.5	0.9	1.1	0.1	51.3	3.6	2.4	0.2
SSV	Super slim	100	17	11.0	0.6	0.8	0.0	37.1	1.3	2.1	0.1
SSC	Super slim, charcoal filter	100	17	11.6	0.7	0.8	0.1	32.7	2.2	1.8	0.1
SSMIX	Super slim, mixed blend^b^	100	17	8.2	0.4	0.6	0.0	35.7	2.2	1.7	0.1

*Note.* ^a^ISO = International Organization for Standardization smoking condition; ^b^ = Health Canada Intense smoking condition; ^c^ =  PM =  Total Particulate Matter; ^d^Mixed blend refers to a tobacco blend of different tobacco types.

### Cigarette Smoke Condensates (CSCs)

CSCs were prepared by Labstat International ULC (Kitchener, ON, Canada). The cigarettes were conditioned [29] and smoked using the International Organization for Standardization (ISO) smoking regime [30], as well as the Health Canada Intense (HCI) smoking regime [31]. Briefly, the ISO smoking regime uses a puff volume of 35 mL, puff duration of 2 s, puff interval of 60 s, and no blocking of ventilation holes. The HCI method employs more intense smoking conditions with a puff volume of 55 mL, puff duration of 2 s, and puff interval of 30 s and 100% blocking of ventilation holes. Approximately 300 mg total particulate matter (TPM) was collected onto Cambridge filter pads (92 mm) and extracted into dimethylsulfoxide (DMSO) to provide a final concentration of ∼10 mg TPM/mL. The CSCs were stored at −20°C prior to use.

### Dilution series

For toxicity measurements, each CSC was initially tested across a twofold serial dilution between 0.725 µg/mL and 100 µg/mL in HEPES buffered E3 media (10 mM HEPES pH 7.2, 5 mM NaCl, 0.17 mM KCl, 0.33 mM CaCl_2_•2H_2_O, 0.33 mM MgSO_4_). Following the initial assessment, concentration ranges were narrowed to represent a range between the highest concentration with no observable effect and the lowest concentration that lead to lethality or the maximum concentration allowable based on carrier controls. Lethality was defined as larvae that lacked cardiac function and were unresponsive to touch, or were in a state of decomposition.

### Developmental Toxicity

At 6 hours post-fertilization (hpf), embryos were loaded into a 96 well plate, 1 embryo/well with 150 µL HEPES E3/well using a large bore micropipette tip created by cutting off the tip. A 2X solution of a single CSC was subsequently added to each well. Plates were then placed in an incubator at 28°C and assessed at 72 hpf for both hatch rate and developmental abnormalities. Replicate experiments were run on separate days (n = 12/day).

### Cardiac Toxicity

Larvae were dechorionated and loaded into 96 well plates between 24 & 26 hpf and treated with a single CSC as described above. Heart rates were measured between 48 & 50 hpf following 24 hours of exposure for 3 embryos/concentration/replicate. Replicate experiments were run on separate days. Significant differences between dilutions were determined through a 1-way ANOVA followed by a Dunnet's post-hock test comparing each dilution to controls.

### Behavioural Toxicity

Larvae were loaded into 96 well plates at 72 hpf and treated with a single condensate as described above. Larval activity was assessed between 120 and 126 hpf by video tracking with a Viewpoint Life Sciences Zebrabox and their activity was monitored using the Viewpoint video tracking system and software (Viewpoint Life Sciences Inc., Montreal, QC, Canada). The plate temperature was maintained in the Zebrabox chamber at 28°C by partial immersion in a recirculating water bath. All experiments consisted of 20 min of acclimation in the dark followed by 4–10 min cycles containing a 5 min light and 5 min dark phase (60 min total). Changes in activity were analyzed using GraphPad-Prism software. Duplicate dilution series experiments were run on two separate days (n = 12/concentration/day).

### Acute Toxicity

Acute toxicity was measured for treatments between 72 & 120 hpf without condensate replacement following behavioural tracking using the same lethality parameters as described above.

### Data Analysis

In order to compare individual CSCs, concentration-response curves were generated for each toxicity measurement (except cardiac toxicity) using GraphPad-Prism software. Teratogenicity and lethality curves were constrained between 0 and 100%. For hatch-rate, curves were constrained at 0, since the maximum hatch rate of controls at 72 hpf was approximately 75–80%. For the behavioural assays, since larvae increase their activity in response to a transition from light to dark, in order to measure the effect of compounds on this inducible activity level, the light activity was considered to be the baseline level and was subtracted from the dark activity for each cycle. The activity during the 4 cycles was then pooled for further analysis. Concentration-response curves were generated by normalizing the dark response at each concentration to the average response of carrier control larvae for each run and fitted by constraining the bottom to 0. For all measures of toxicity an f-test was used to compare log EC/LC/IC_50_ and Hill slope values between CSCs in order to rank the toxicity profiles.

### Quantitative Polymerase Chain Reaction (qPCR)

72 hpf embryos were transferred to 6-well plates at a density of 10 embryos/well in E3 (5 mM NaCl, 0.17 mM KCl, 0.33 mM CaCl2•2H2O, 0.33 mM MgSO4). The media was replaced with 3 mL of HE3, and then spiked with CSC solution to obtain a concentration equal to the LD_80_ as determined in the 72–120 hpf toxicity assay. DMSO (0.1%) was used as control. After 5.5 h embryos were examined microscopically to ensure no death had occurred, and three wells of either treated or DMSO control fish were pooled. Embryos were anesthetized on ice, frozen on dry ice, and then stored at −80°C until processing. Embryos were thawed on ice, and then RNA was isolated using a Total RNA Purification Kit (Norgen Biotek, ON, Canada) following the manufacturer's instructions for animal tissues with the following modifications: 30 embryos per tube were homogenized by pipetting in 500 µl of lysis buffer containing 1% β-mercaptoethanol (Sigma, ON, Canada), and spun for 5 min at RT, ∼14k x g. After ethanol addition, samples were spun for 2 min, RT, ∼14k x g. The optional on-column DNaseI digestion was performed using RNase-Free DNaseI (Norgen Biotek, ON, Canada). Finally, RNA was eluted in 30 µL of elution buffer. The concentration and purity of the RNA was then determined spectrophotometrically, and 1 µg of RNA was used to make cDNA using Superscript III reverse transcriptase (Invitrogen, ON, Canada) accordingly. No-RT added controls were performed on all RNA samples. All cDNA was stored at −20°C until use. All qPCR reactions were performed in 10 µL volumes in 384 well PCR plates using a Roche LightCycler thermalcycler (Roche, QC, Canada). Each reaction received 4 µL of cDNA (diluted 1/15 with deionized water (Sigma, ON, Canada), and 6 µL of enzyme/primer premix (5 ul of 2x KAPA SYBR green (KAPABiosystems, ON, Canada), 0.25 µL of each 10 mM primer (S1 **Table**), and 0.5 mL of deionized water (Sigma, ON, Canada). The program consisted of 45 cycles of amplification, an annealing temperature of 60°C, followed by a melt curve. Three technical replicates were run for each primer pair and cDNA per plate, and three biological replicates were performed for each compound. Data was analyzed accordingly as follows: technical repeats were assessed (melt curve, Ct) and averaged. The average Ct of the housekeeping gene (EF-1a) was then subtracted from the target gene Ct (delta Ct). As a control, Rpl13 transcript was also measured to validate the use of the EF-1a as a housekeeping gene. Expression and melt curves were assessed for all wells, and manually edited to remove problematic wells. Technical repeats were averaged, and then the housekeeping gene subtracted (delta Ct). A Student's t-test (2 tailed, unpaired, equal variance) was then performed on the three biological replicates for each gene separately, with p <0.05 being considered significant. For graphing, data were transformed using the formula 2ˆ(−delta Ct), and normalized to the appropriate carrier control. The specificity of each primer pair was initially evaluated by performing a BLAST comparison to the zebrafish genome. The primer pair specificity was subsequently tested by running the product of the qPCR reaction on a 1% agarose gel to ensure a single amplicon followed by gel extraction and sequencing of the amplicon. For primer pairs and gene accession numbers see S1 Table.

## Results

### Developmental Toxicity/Teratogenicity (6–72 hpf)

**Figure 1:**
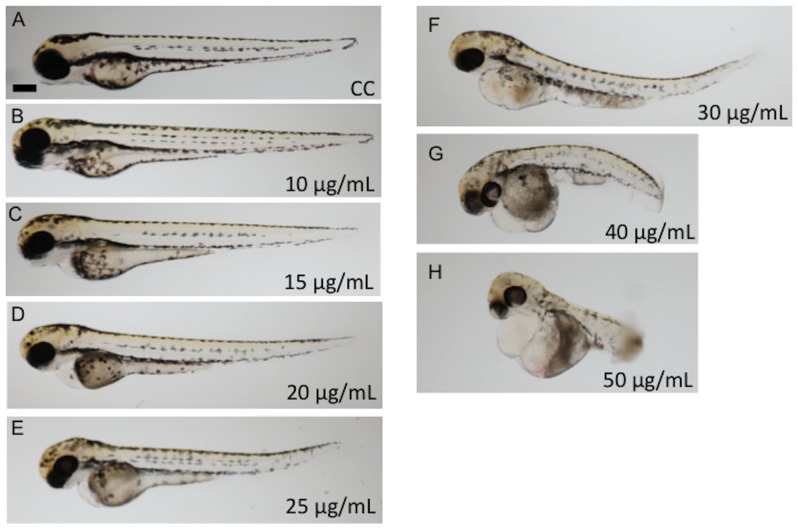
Concentration response profiles of zebrafish larvae exposed to MBV condensate from 6 to 72 hpf. **A**) DMSO Carrier control (CC); **B-H**) 10, 15, 20, 25, 30, 40, 50 µg/mL CSC, respectively. Scale bars correspond to 280 µm.

**Figure 2:**
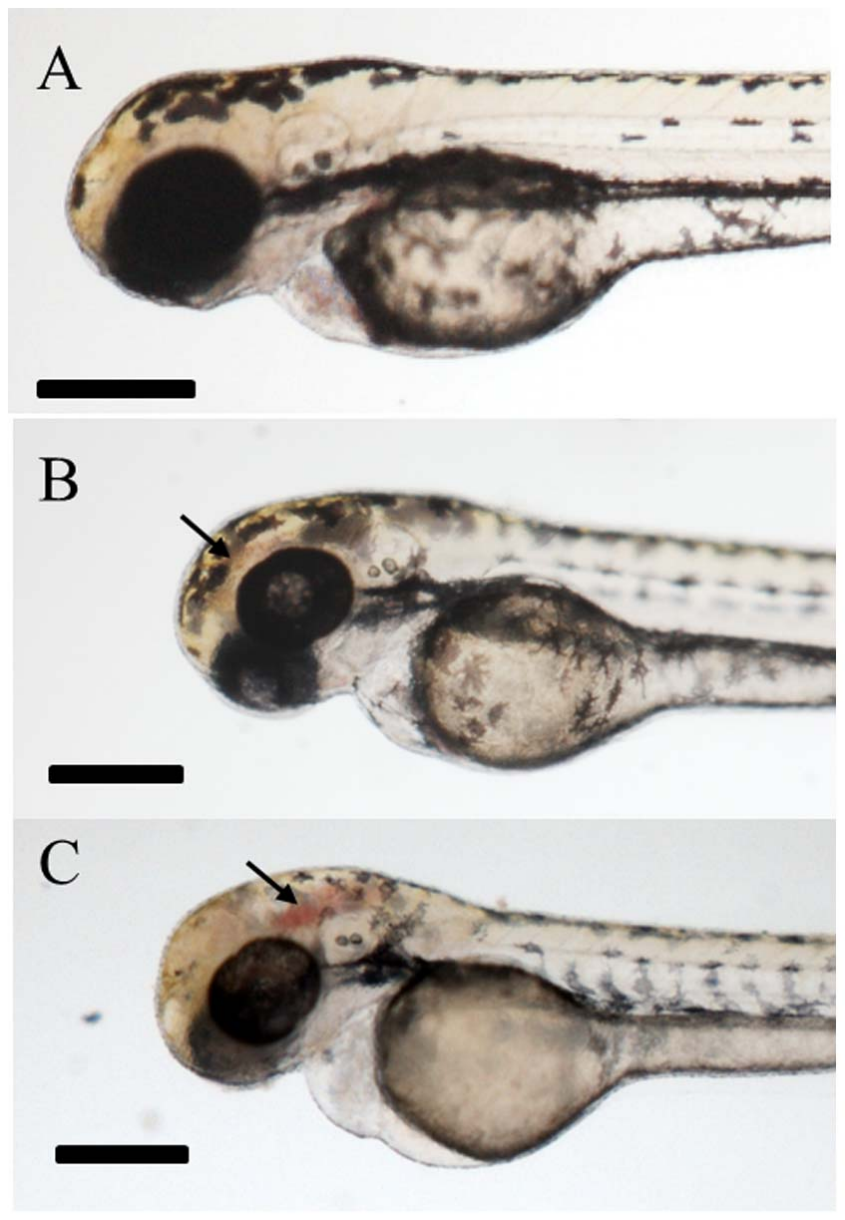
Cerebal hemorrhaging in zebrafish larvae exposed to BSV condensate from 6–72 hpf. **A**) DMSO Carrier control (CC); **B**) 12.5 µg/mL CSC. **C**) 20 µg/mL CSC. Arrows indicate blood pooling. Scale bars correspond to 280 µm.

The developmental toxicity/teratogenicity of each CSC was tested by continuous exposure of zebrafish embryos to an individual CSC, at a number of concentrations, from 6 to 72 hpf. A concentration dependent increase in the phenotypic abnormalities associated with each of the CSCs tested was apparent (**Fig. 1**). These abnormalities included, but were not limited to: developmental delay, malformed head and eyes, truncated tail, pericardial edema, curvature of the body and/or hypo-pigmentation (reduction in the melanophore numbers or pigment level). CSC exposure also led to cerebral hemorrhaging beginning at 48 hpf, coinciding with the time of major blood vessel restructuring and angiogenesis within the head (**Fig. 2**).

In order to calculate EC_50_ values and to compare toxicity levels between each CSC, the percentage of larvae that showed one or more phenotypic abnormalities was used to generate a concentration-response curve based on the percentage of affected larvae at each concentration. As shown in **Table 2** **&** **3**, there were overlaps in the toxicity levels for some of the CSCs. Specifically, for CSCs prepared using the ISO smoking regime, MIX was found to be most toxic, followed by SSMIX, then BSV, CM8, MBV, 3R4F and SSV as a group, followed by SSC (**Table 2**). The rankings were similar but not identical for CSC prepared under the HCI smoking conditions, with MIX being the most toxic followed by MBV, then 3R4F, CM8, BSV and SSMIX as a group, and finally SSC and SSV as a group (**Table 3**). When examining the impact of smoking condition on CSC toxicity, it was found that the use of ISO smoking regime resulted in CSCs that were, in general, more toxic than those obtained under HCI conditions. The exceptions were CSCs from MIX and MBV, where no significant differences in teratogenicity were observed (**Table 2** **&** **3**).

**Table 2:** Comparison of the toxicity profile of zebrafish larvae following exposure to cigarette smoke condensates (CSCs).

Identifier	Teratogenicity		Identifier	Hatch Rate		Identifier	Lethality		Identifier	Behaviour	
	EC50	SEM		EC50	SEM	Brand	LD50	SEM	Brand	EC50	SEM
MIX	10.9	1.03	*3R4F	16.9	1.07	MIX	26.5	1.01	MIX	22.3	1.05
SSMIX	13.3	1.04	*MIX	17.1	1.12	*MBV	36.7	1.02	*MBV	31.7	1.08
*BSV	15.0	1.06	*MBV	17.5	1.32	*BSV	37.9	1.01	*SSMIX	35.0	1.07
*CM8	16.5	1.03	*SSMIX	20.3	1.07	*CM8	38.7	1.02	*CM8	36.2	1.04
*MBV	17.4	1.04	*BSV	22.2	1.07	SSMIX	40.3	1.01	*BSV	36.2	1.05
*3R4F	17.6	1.04	*CM8	24.9	1.06	*SSV	45.3	1.02	3R4F	40.0	1.02
*SSV	20.4	1.07	SSV	32.3	1.06	*3R4F	45.5	1.01	SSV	43.2	1.03
SSC	23.0	1.03	SSC	32.5	1.12	SSC	52.3	1.01	SSC	49.9	0.00

Concentration response (ug/ml) of CSCs prepared using the International Organization for Standardization (ISO) smoking regime and CSCs prepared using the Health Canada Intense (HCI) smoking regime. Asterisk indicates no significant difference between cigarette types for the specific toxic parameter measured (f-test, p>0.05).

**Table 3:** Comparison of the toxicity profile of zebrafish larvae following exposure to cigarette smoke condensates (CSCs).

Identifier	Teratogenicity		Identifier	Hatch Rate		Identifier	Lethality		Identifier	Behaviour	
	EC50	SEM		EC50	SEM		LD50	SEM		EC50	SEM
MIX	11.5	1.03	*3R4F	22.1	1.38	MIX	26.7	1.01	MIX	22.9	1.06
MBV	18.4	1.02	*MIX	23.4	1.16	MBV	46.1	1.03	MBV	40.5	1.02
*3R4F	20.6	1.04	*CM8	24.5	1.18	CM8	49.0	1.01	CM8	43.9	1.03
*CM8	21.8	1.06	MBV	27.6	1.08	*SSC	52.9	1.02	BSV	49.0	1.05
*BSV	22.1	1.02	*BSV	40.8	1.07	*SSMIX	56.1	1.02	3R4F	54.7	1.04
*SSMIX	23.5	1.03	*SSMIX	41.2	1.05	SSV	62.1	1.01	*SSC	60.8	1.02
*SSC	27.4	1.03	*SSV	49.0	1.06	*BSV	70.1	1.02	*SSMIX	61.7	1.03
*SSV	28.4	1.02	SSC	65.5	1.05	*3R4F	71.7	1.03	SSV	62.6	1.04

Concentration response (ug/ml) of CSCs prepared using Health Canada Intense (HCI) smoking regime. Asterisk indicates no significant difference between cigarette types for the specific toxic parameter measured (f-test, p>0.05).

### Chromatophore response (48–72 hpf)

**Figure 3:**
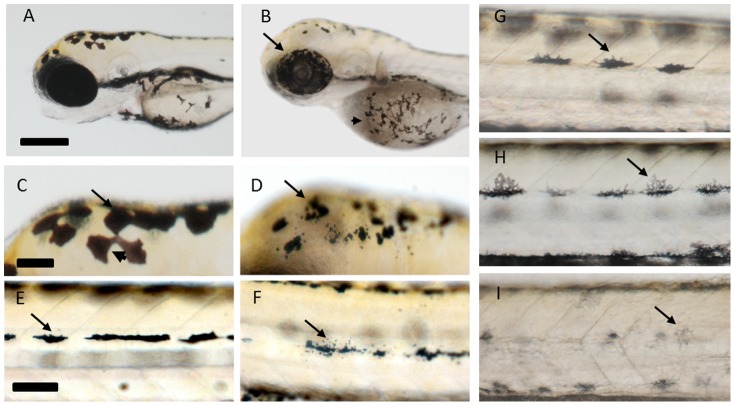
Changes in melanocyte patterning and distribution following cigarette smoke condensate (CSC) exposure from 48–72 hpf. A, C, E, G) DMSO Carrier control (CC). Changes in pigment distribution (arrows) within the melanphores of the eyes and yolksac (B), dorsal regions of the head (D) and the tail midline (F) following treatment with 50 µg/mL CSC prepared from 3R4F using the ISO smoking regime, are evident. Concentration-dependent change in melanocyte morphology and pigment level (arrows) following exposure to CSC prepared from the Canadian best seller (BSV) using the ISO smoking regime (H-6.1 µg/mL; I-50 µg/mL). Scale bars in A, C and E correspond to 280, 70 and 80 µm, respectively.

CSC exposure from 6–72 hpf revealed a number of adverse effects on the developing embryo include phenotypic changes to the melanocytes. To address the consequence of CSC exposure on melanocyte patterning, morphology and pigment levels we narrowed the window of exposure to 48–72 hpf. This reduced the impact of earlier developmental effects so we could better evaluate the consequence of exposure on these cells. The larvae were treated with CSCs from 3R4F and BSV prepared under ISO conditions (Fig. 3) which resulted in phenotypic changes to the melanocytes that were similar to that observed in other species in response to toxic insults [33].

### Hatching (6–72 hpf)

In addition to the assessment of gross morphological changes, larval hatch rate was also measured between 6 and 72 hpf on the same cohort of larvae. The assessment of larval hatch rate is a standard toxicity measurement of zebrafish larvae. Hatching requires secretion of proteolytic enzymes from the hatching gland to soften the chorion and larval movement to break free. In the absence of overt developmental perturbations a change in the hatch rate would suggest a disruption in one of these processes. Under control conditions at 72 hpf 75–80% of larvae hatch. Similar to the developmental toxicity profiles, a concentration response curve was generated in order to compare the individual CSCs and a noticeable overlap in the effect of the individual CSCs on hatch rate was observed (**Table 2** **&** **3**). The CSCs prepared using the ISO smoking regime were divided into two different groups that were not statistically different. CSCs from 3R4F, MIX, MBV, SSMIX, BSV and CM8 were more toxic than CSCs from SSV and SSC. For the CSCs prepared under HCI conditions, 3R4F, MIX and CM8 were found to be the most toxic, followed by MBV, then BSV, SSMIX and SSV as a group, followed by SSC. With regard to the impact of smoking regime on hatch rate, no significant difference in the hatch rate was observed for CSCs prepared from the two reference cigarettes (3R4F and CM8). However, for the commercial cigarettes, CSCs prepared using the ISO smoking regime had a greater effect on hatch rate than those prepared under HCI conditions for 5 of 6 brands tested (MBV, BSV, SSMIX, SSC & SSV).

### Acute toxicity (72–120 hpf)

By 72 hpf the developing zebrafish enters the larval stage, at which time body patterning is largely established and the adverse effects of test substances are no longer considered to be entirely developmental in nature. The acute toxicity of each CSC was tested between 72 and 120 hpf under conditions of constant exposure to a single CSC at a range of concentrations with no replacement. Lethality was defined as larvae that lacked cardiac function and were unresponsive to touch or in a state of decomposition. As shown in **Table 2**, when ISO smoking conditions were used, the CSC from MIX was found to be most toxic followed by MBV, BSV and CM8 as a group, SSMIX, then SSV and 3R4F, and finally SSC as the least toxic. For CSCs prepared under HCI conditions (**Table 3**), MIX was once again found to be most toxic followed by MBV, CM8, then SSC and SSMIX as a pair, SSV and finally, BSV and 3R4F as a pair. When comparing the two different smoking methods, CSCs from both of the reference cigarettes (3R4F and CM8) along with 5 of 6 of the commercial cigarettes (MBV, BSV, SSMIX, SSC, SSV) all showed greater lethality when prepared under ISO conditions (**Table 2** **&** **3**).

### Behavioural toxicity (72–120 hpf)

**Figure 4:**
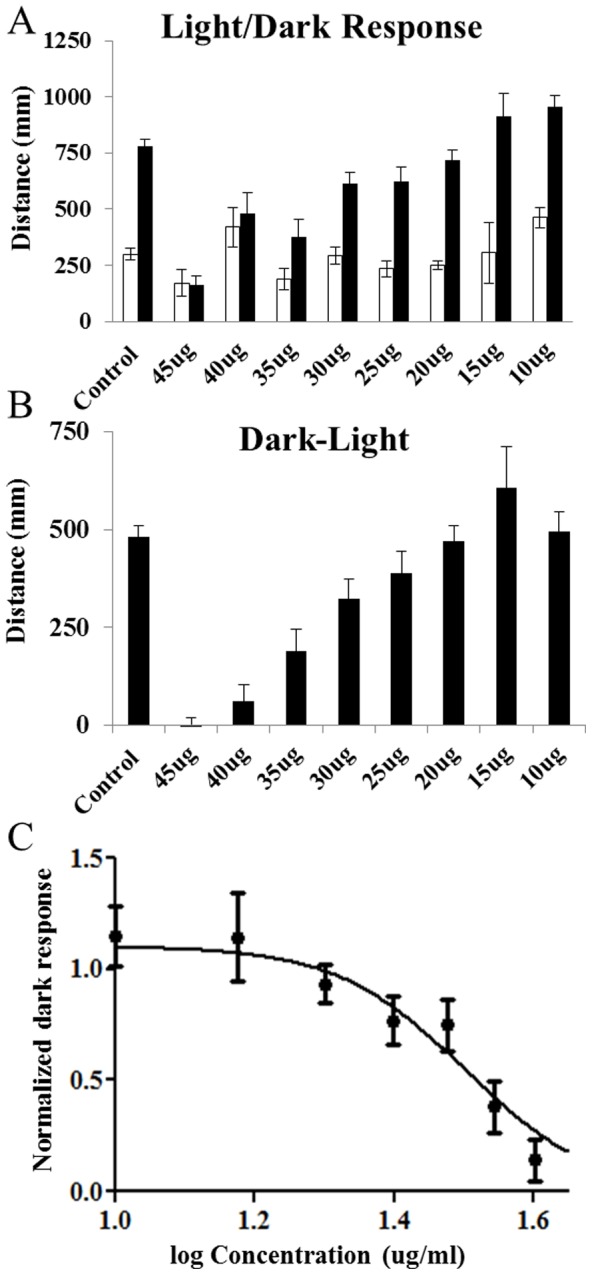
Behavioural toxicity following exposure to the MBV condensate from 72–120 hpf. A) Larval response to 5 minute cycles of light-dark. B) Dark startle response presented as the difference between the light and dark response levels. C) Concentration response curve for dark response.

At 120 hpf zebrafish larvae display innate, quantifiable patterns of behaviour that can be manipulated by visual stimuli and chemical treatment. When subjected to short periods of darkness zebrafish larvae display a stereotypical avoidance response that leads to an increase in activity (**Fig. 4**). Larvae were tested for changes in this normal response pattern following exposure to each CSC from 72–120 hpf at a range of concentrations based on the acute toxicity values. The light activity level was considered the baseline activity and the larval dark-response for each compound was obtained by subtracting this from the activity during the dark periods. These values were subsequently plotted as a fraction of control activity in order to generate a concentration-response curve (**Fig. 4**). In general, exposure to the CSCs led to a concentration-dependent reduction in response to a transition from light to dark. For the CSCs prepared under ISO conditions MIX was found to have the greatest effect on activity, followed by MBV, SSMIX, CM8 and BSV as a group, then 3R4F, SSV and SSC (**Table 2**). Under HCI conditions, CSC from MIX once again caused the largest effect on activity, followed by MBV, CM8, BSV, 3R4F as a group, then SSC and SSMIX as a pair, and finally SSV (**Table 3**). CSCs prepared using the ISO smoking regimes had a greater effect on behaviour than CSCs prepared under HCI smoking conditions for all the cigarettes tested.

At concentrations that do not produce lethality or overt phenotypic changes, an alteration in the normal behavioural response pattern may be the result of either a change in neuromuscular function or neurotoxicity. When the larval activity was normalized to control levels it was possible to statistically compare the IC & LC_50_ values for each CSC (f-test of log curves). Of the CSCs that were studied, only the CSC prepared from MBV under HCI conditions showed a significant difference in the log-IC/LC_50_ levels. This suggests that any behavioural toxicity produced by the CSC (excluding MBV), as measured by a change in activity, is accompanied by general toxicity.

### Cardiac Toxicity (24–48 hpf)

Cardiac toxicity was assessed by manually counting larval heart rates in response to exposure to an individual CSC at a number of concentrations following exposure from 24 to 48 hpf. This exposure regime was chosen in order to maximize exposure to the CSCs between manual dechorination at 24 hpf and scoring of the heart rates while larvae remain in a natural lateral recumbent position without anesthesia which can affect heart rate. At concentrations where there was no lethality, heart rates were reduced by less than 50% for all of the CSCs tested. This prevented the calculation of an IC_50_ value using a non-linear regression method similar to that employed for the lethality and behavioural toxicity analysis. Therefore, the effect on cardiac function was instead assessed by identifying the concentration of each CSC that resulted in a significant reduction in heart rate and by determining the reduction in heart rate at 50 µg/mL (the maximum concentration of CSC tested). This assessment allowed for a qualitative comparison of the CSCs and revealed that CSCs prepared from MBV, CM8 and SSMIX under ISO conditions, as well as CSC from CM8 prepared under HCI conditions, were the most potent and led to significant reduction in heart rate at 15 µg/mL. The CSC prepared from SSMIX under HCI conditions appeared to have the least effect on cardiac function, producing a significant reduction in heart rate at concentrations of 40 µg/mL (**Table 4**).

**Table 4:** Changes in heart rate following larval exposure to cigarette smoke condensates (CSCs) from 24–48 hpf.

SAMPLE ID	ISO		HCI	
	Minimum Significant Reduction (bpm)	Maximum Significant Reduction (bpm)	Minimum Significant Reduction (bpm)	Maximum Significant Reduction (bpm)
3R4F	11.5^a^	24.5^b^	10.8^a^	17.2^b^
CM8	12.0^d^	27.0^b^	13.0^d^	21.5^c^
BSV	14.7^c^	32.0^b^	13.7^e^	27.0^b^
MIX	7.5^e^	39.7^b^	7.9^a^	37.2^b^
MBV	9.0^d^	36.0^b^	16.4^e^	32.0^b^
SSV	15.0^f^	19.5^b^	11.0^c^	22.0^b^
SSC	11.9^c^	19.2^b^	10.2^f^	15.7^b^
SSMIX	8.9^d^	36.7^b^	8.4^b^	8.4^b^

CSCs were prepared under the International Organization for Standardization (ISO) and Health Canada Intense (HCI) smoking regimes. bpm = beats per minute.

*Note*. Concentrations of CSC: ^a^25 µg/mL; ^b^50 µg/mL; ^c^30 µg/mL; ^d^15 µg/mL; ^e^20 µg/mL; ^f^40 µg/mL.

### Molecular Mechanisms of Toxicity

In order to evaluate whether the zebrafish model can be used to identify smoking-related gene expression changes caused by exposure to the CSCs and to identify possible molecular mechanisms of toxicity, a number of gene targets were selected from humans, higher vertebrates and zebrafish that are associated with inflammatory responses, metabolism, cancer, development, cell cycle control, cellular stress and toxicity and were used to evaluate changes in gene expression in response to CSCs.

Initially the transcript levels of 112 genes were assessed following exposure to the CSC prepared from MBV under ISO conditions by quantitative PCR (qPCR). Larvae were exposed from 72–77 hpf to a single concentration of the selected CSC corresponding to the LC_80_ derived from the acute toxicity experiments (72–120 hpf). These concentrations and times were selected to identify the gene expression changes, prior to lethality, in a group of larvae where the majority were expected to succumb to the treatment. This data was used to identify a subset of informative target genes that could be utilized for testing the other CSCs. Following the initial assessment a testing panel of 28 genes was selected based on: (1) genes that were found to change expression levels in the initial analysis of CSC from MBV, (2) genes linked to cancer, (3) genes known to be differentially expressed in response to cigarette smoke and (4) genes potentially related to specific phenotypic readouts (pigmentation, blood pooling, cardiac effects) (**Table 5**). Changes in the expression of this select set of genes were evaluated following larval exposure to CSC prepared from MBV using both the ISO and HCI smoking regimes, in order to study the impact of the two different smoking regimes on the patterns of gene expression. Significant changes were found for 16 of the 28 genes following exposure to the CSCs prepared using either the ISO or the HCI smoking regime (**Table 5**). Of the 16 genes where the expression was altered, 11 were found to change for the CSC prepared under both ISO and HCI, while the other 5 were found to change for only one of the two smoking conditions. Therefore, CSCs produced under the two smoking regimes appear to alter the expression of a similar set of genes, but at different levels.

**Table 5:** Gene expression changes (fold change) following larval exposure to the MBV condensate from 72 to 77 hpf as measured by qPCR.

Gene	ISO		HCI	
	Average	SEM	Average	SEM
ahr and detox				
Acylcoa	1.51	0.2	2.80*	0.2*
ahrr1	82.33*	2.6*	70.37*	1.3*
ahr2	2.94*	0.3*	4.59*	0.3*
cyp1a1	271.83*	42.7*	398.54*	37.1*
cyp1b1	35.48*	15.6*	39.73*	3.9*
cyp2aa12	4.96*	0.3*	7.48*	0.6*
cyp2u1	1.22	0.05	1.72*	0.2*
Gnmt	1.33	0.1	1.67	0.2
mat1a	0.52	0.06	0.74	0.1
Regulators				
chk2	0.31*	0.07*	0.74	0.05
cox2	3.07	0.7	2.23	0.5
Fos	2.34	0.3	4.23	0.6
klf2a	0.39*	0.02*	0.54*	0.02*
nos1	0.68	0.2	1.39	0.2
pak2	0.45*	0.02*	0.72	0.04
Vegfaa	1.37	0.3	2.28	0.2
Apoptosis				
bcl2	2.54*	0.2*	3.87*	0.4*
casp8	1.23	0.08	1.96*	0.1*
ddit4	1.20	0.08	1.52	0.4
gadd45al	3.92*	0.9*	5.16*	1.9*
hsp70	26.57*	2.3*	36.21*	3.1*
il-1b	1.58	0.2	2.26	0.6
Pigment				
Cbsa	0.5	0.01	0.52	0.08
Cbsb	1.94*	0.05*	2.28*	0.2*
Mitfa	0.24*	0.02*	0.37*	0.03*
Pmch	1.08	0.1	1.44	0.3
Pomc	0.96	0.1	2.35	0.7
Tyr	0.73	0.1	1.25	0.09

Significant changes in gene expression are indicated with an asterisk. Cigarette some condensates were prepared under the International Organization for Standardization (ISO) and Health Canada Intense (HCI) smoking regimes.

## Discussion

By using a multifaceted approach for evaluating toxicity we have shown that zebrafish embryos and larvae can be used to study the adverse effects associated with CSC exposure by evaluating developmental, cardiac function, behavioural/neural activity and acute toxicity.

Research and epidemiological studies have highlighted the adverse health effects associated with cigarette smoke exposure including the negative impacts during embryonic development. Smoking during pregnancy is known to cause negative outcomes such as fetal growth restriction, low birth weight, premature births [1] and can affect the development of nervous, respiratory and cardiovascular systems [34]. We have shown that exposure of zebrafish embryos to CSCs from 6 to 72 hpf also affects normal development leading to body axis malformations, head and trunk abnormalities and significant developmental delay. This suggests that both cigarette smoke and CSCs share similar organismal toxicity profiles during vertebrate embryonic development. In addition, numerous reports have demonstrated the utility of using the zebrafish model for assessing cardiac toxicity in response to exposure to a range of chemicals and complex mixtures [35]–[40]. The reduction in embryonic heart rates following exposure to CSCs may indicate the potential to negatively impact cardiac function.

A number of interesting toxicity profiles were found when comparing the CSCs from the different cigarettes studied. One of the most prominent patterns that emerged was the finding that for nearly all of the toxicity tests, CSCs generated under ISO conditions were more toxic than those prepared under HCI conditions. These observations are consistent with previous cell based-studies of the mutagenic and cytotoxic potency of CSCs which report that CSCs prepared under more intense smoking regimes are less toxic than CSCs prepared using the ISO smoking regime [41]–[43]. While the precise reasons for this are unknown, it has been proposed that differences in smoke chemistry along with more complete combustion and greater production of water with the use of more intense smoking regimes results in CSCs that are less toxic than those prepared under ISO conditions.

In addition to differences in toxicity between the smoking regimes we have also shown that the zebrafish model can be used to study the impact of cigarette design on CSC toxicity. There were some notable differences in the toxicity profiles of the CSCs that appeared to be linked to specific cigarette design features. Most notably, CSCs from the super slim cigarettes (SSV and SSC) were the least toxic in many of the assays. It also appears that cigarettes containing a blend of tobacco as opposed to a single tobacco type may influence CSC toxicity. While more work is required to evaluate which cigarette characteristics have the greatest influence on the toxicity profiles of individual condensates, we have shown that the zebrafish model can provide *in vivo* data for evaluating the adverse effects associated with different tobacco products, identifying those that warrant further investigation.

In addition to the phenotypic and behavioural changes induced by exposure to the CSCs, gene expression changes were also apparent. We have shown that a number of gene categories that are altered by cigarette smoke exposure in mammalian systems are also altered in intact zebrafish larvae exposed to CSCs. Out of the 112 gene targets tested using qPCR, a number of different groups were shown to be affected in response to CSC exposure including genes involved in cell cycle, apoptosis, developmental regulation, aryl hydrocarbon receptor signaling and detoxification pathways (**Table 5**). Apoptotic markers such as caspase-8 were upregulated by the CSC from MBV prepared using the HCI smoking conditions, which correlates with caspase-8 activation in mice in response to mainstream cigarette smoke [44] and in rat cardiac tissue in response to second hand smoke [45]. Exposure to the CSCs from MBV prepared using both smoking conditions also led to the induction of genes that are associated with the activation of the Aryl hydrocarbon (AH) and detoxification pathways. Elevated expression of genes involved in the detoxification and AH pathways resemble the changes observed in the transcriptome of exposed bronchial and nasal epithelial tissue in response to cigarette smoke in humans [46], [47] suggesting the effects of CSC exposure in zebrafish larvae mirror the effects of cigarette smoke exposure in other systems not only at the level of overt toxicity but also at the molecular level.

The changes in gene expression associated with CSC exposure have revealed that the zebrafish model can potentially be used to uncover the underlying molecular changes associated with tobacco exposure that cannot be identified with cell line based systems. Larvae exposed to CSCs at 72 hpf also led to a decrease in Krüppel-like factor 2a (Klfa) gene expression. In zebrafish klf2a is expressed in cells closely associated with many of the blood vessels in the head and trunk and is involved in regulating angiogenesis, cardiac development and hematopoiesis [48]. Although, the Klf gene family has been implicated in a number of processes during normal embryonic development the reduction in expression in response to CSC exposure provides a potential mechanism for the cerebral hemorrhaging that may be conserved in higher vertebrates. The Klf gene family plays similar roles in mammals including regulating blood vessel stability, erythroid cell maturation and T-cell activation. Interestingly, klf2 knockout mice succumb from massive hemorrhaging in utero [45], [49]–[51] suggesting that CSC exposure in zebrafish embryonic development may interfere with vascular stability through altering klf2 function.

## Conclusions

Overall it is now apparent that the zebrafish toxicity models developed in this study are capable of producing a complex, multifaceted profile that can be used to assess the toxicity associated with cigarette smoke condensates. This model extends the data generated from the *in vitro* and cell culture models currently employed for cigarette toxicity testing and could be useful in probing the changes in organismal toxicity relating to cigarette design which is not apparent in cell culture assays. Interestingly, the toxicity profiles of the different cigarettes studied showed large overlaps and the toxicity levels are generally within a narrow concentration range. This suggests that CSCs from the different cigarettes are somewhat comparable in terms of their toxicity and cigarette design features such as alternate filters and the super slim circumference only result in small differences in CSC toxicity. While our results indicated that the zebrafish larvae can be used to compare CSCs from cigarettes with different design features, it must be emphasized that human exposure and smoking behavior were not examined and the results from this study cannot be directly translated to human health impact. Therefore, cigarettes with a lower CSC toxicity profile cannot be considered a ‘reduced harm’ cigarette. However, the overlaps between the toxicity profiles found in the current study and those previously found for mammalian models suggests that the response of zebrafish larvae to CSC exposure can be linked to other models of disease. A more detailed assessment of the chemical differences between the condensates derived from different cigarette types and the toxicity profiles produced may provide insights into the components of cigarette smoke that are most likely to produce disease. Importantly this model provides a platform with which to contrast both a larger number of CSCs and the chemical constituents found therein.

## Supporting Information

S1 Table **Primer sets used for quantitative PCR experiments.**
(DOCX)
