# Supplementary material for: Use of the Zebrafish Larvae as a Model to Study Cigarette Smoke Condensate Toxicity
Source: PLoS One. 2014 Dec 19;9(12):e115305. doi: 10.1371/journal.pone.0115305 (PMC4272283; doi:10.1371/journal.pone.0115305)
Supplement: S1 Table — Primer sets used for quantitative PCR experiments. (DOCX) [file pone.0115305.s001.docx]

**Table S1.** Primer sets used for quantitative PCR experiments.

| **Gene symbol** | **Name and alias** | **Genbank ID** | **Primer name** | **Primer sequence (5' -3')** |
| --- | --- | --- | --- | --- |
| vegfaa | vascular endothelial growth factor Aa (Danio rerio); vegf; vegfa; wu:fj82c06; vegfaa | NM_131408.3 | VEGFAA F1 | GTGCAGGATGCTGTAATGATGAGGCG |
|  |  |  | VEGFAA R1 | GAGCAAGGCTCACAGTGGTTTTCTT |
| Pak2 | p21 (CDKN1A)-activated kinase 2a [ Danio rerio ];pak2; cb422; kinase; zgc:91798; wu:fb71h01; si:dz198m22.1; pak2a | NM_001002717.1 | DrPak2-F2 | AGAGCCTCTATCACTCCAGCAG |
|  |  |  | DrPak2-R2 | GGTCTACAATCTGTGGCTCCTC |
| Klf2a | Kruppel-like factor 2a [ *Danio rerio* ],fb08e06; wu:fb08e06; klf2a | NM_131856.2 | Klf2a-F1 | GAGGAAACGCACGGCGACTCAC |
|  |  |  | Klf2a-R1 | GGTCGGTGTCCGGTGTGCTTG |
| DDIT4 | DNA-damage-inducible transcript 4 [D. rerio]; ddit4; sb:cb821 | NM_200107.1 | zDDIT4_F | ACGCAGCGGTTTAGCTTACTTG |
|  |  |  | zDDIT4_R | GCATGGAATATGCATGTGATTG |
| acyl-coAsyn | Danio rerio acyl-CoA synthetase long-chain family member 4a (acsl4a), mRNA | NM_200649.1 | acylcoAsynF | CCTCTGATCTGCTGTGAGGTC |
|  |  |  | acylcoAsynR | GATGAGGCTTGTCACGCTTT |
| AhR2 | Danio rerio aryl hydrocarbon receptor 2 (ahr2), mRNA | NM_131264.1 | AhR2F | CTACTTGGGCTTCCATCAGTCG |
|  |  |  | AhR2R | GTCACTTGAGGGATTGAGAGCG |
| AhR1 | Danio rerio aryl-hydrocarbon receptor repressor a (ahrra), mRNA | NM_001035265.1 | AhR1F | GCGCATCAAGAGCTTCTGCAGCGTGTT |
|  |  |  | AhR1R | CCACTGACGACCAGCGCAAACCCT |
| CYP1A1 | Danio rerio cytochrome P450, family 1, subfamily A (cyp1a), mRNA | NM_131879.1 | CYP1A1F | AATCCCAGACGGGCTACA |
|  |  |  | CYP1A1R | CCGGGCCATAGCACTTAC |
| EF-1a | Danio rerio eukaryotic translation elongation factor 1 alpha 1, like 1 (eef1a1l1), mRNA | NM_131263.1 | EF1A-F2 | GATGCACCACGAGTCTCTGA |
|  |  |  | EF1A-R2 | CGGTCGATCTTCTCCTTGAG |
| RPL13 | Danio rerio ribosomal protein L13a (rpl13a), mRNA | NM_212784.1 | RP-L13-F1 | TCTGGAGGACTGTAAGAGGTATGC |
|  |  |  | RP-L13-R1 | AGACGCACAATCTTGAGAGCAG |
| Casp8 | Danio rerio caspase 8, apoptosis-related cysteine peptidase (casp8), mRNA | NM_131510.2 | zCasp8-F | CTACAGACGCAGAAACTCCAT |
|  |  |  | zCasp8-R | TGTCCATATCAGTGCCTGTTC |
| Chk2 | Danio rerio CHK2 checkpoint homolog (S. pombe) (chek2), mRNA | NM_200045.1 | zchk2-F | CACCATATTTCAACACGTACAGC |
|  |  |  | zchk2-R | TTCCCGTTTCCCAGTTTCTC |
| IL-1b | Danio rerio interleukin 1, beta (il1b), mRNA | NM_212844.1 | zIL-1b F2 | GGCCGTCACACTGAGAGCCG |
|  |  |  | zIL-1b R2 | GAGACGTGGAGCCTTCGGCG |
| Nos-1 | Danio rerio nitric oxide synthase 1 (neuronal) (nos1), mRNA | NM_131660.1 | nos1 F | CTCCATTCAGAGCCTTCTGG |
|  |  |  | nos1 R | CCGACAACCAAACACCAGG |
| v-fos | Danio rerio v-fos FBJ murine osteosarcoma viral oncogene homolog (fos), mRNA | NM_205569.1 | vFos F2 | GTGCAGCACGGCTTCACCGA |
|  |  |  | vFos R2 | TTGAGCTGCGCCGTTGGAGG |
| BCL2 | Danio rerio BCL2 binding component 3 (bbc3), nuclear gene encoding mitochondrial protein, mRNA | NM_001045472.2 | qBCL2For | TGTCTTCCTTCAGAGGAATGCCGT |
|  |  |  | qBCL2Rev | TCCTGTCCTTTGTAACCCGTGTGT |
| GADD45al | Danio rerio growth arrest and DNA-damage-inducible, alpha, a (gadd45aa), mRNA | NM_200576.1 | qGADD45alFor | CATCAACATCCTGCGCGTGAACAA |
|  |  |  | qGADD45alRev | TCTCTCTGCAGAAGCGGTTCACTT |
| cyp1b1 | Danio rerio cytochrome P450, family 1, subfamily B, polypeptide 1 (cyp1b1), transcript variant 3, mRNA | NM_001145708.1 | CYP1B1For | GCTCAGCTGGTCCATTGATACC |
|  |  |  | CYP1B1Rev | CATCAGCGACAGCAACACAC |
| cyp2u1 | Danio rerio cytochrome P450, family 2, subfamily U, polypeptide 1 (cyp2u1), mRNA | NM_001145564.1 | CYP2U1For | CAGGTTTGATAGCGTTCGTACTGG |
|  |  |  | CYP2U1Rev | GAGGGATGTTGGCGTATGTCTG |
| cyp2aa12 | Danio rerio cytochrome P450, family 2, subfamily AA, polypeptide 12 (cyp2aa12), mRNA | NM_001109701.1 | CYP2AA12For | CCAGGTCATAAAGGAAGCCATAG |
|  |  |  | CYP2AA12Rev | CAGTGATCCAGGTTAAAATCGG |
| mat1a | Danio rerio methionine adenosyltransferase I, alpha (mat1a), mRNA | NM_199871.1 | mat1aFor- | GTTGCTGGCAAACATGGAAGGACT |
|  |  |  | mat1aRev- | TACTTTAGCATCCGGGTCCTGCTT |
| gnmt | Danio rerio glycine N-methyltransferase (gnmt), mRNA | NM_212816.1 | gnmtFor | TGATTGAGGAGGCCAACTGGCTAA |
|  |  |  | gnmtRev | AGGCGAGCTTCTGATCACTTTGGT |
| cbsb | Danio rerio cystathionine-beta-synthase b (cbsb), mRNA | NM_001014345.2 | cbsbFor | AGCCAAAGAGCTTAAGGAAGGCCA |
|  |  |  | cbsbRev | AGGCGAAGCTCCTGAAGAGTCAAA |
| cbsa | Danio rerio cystathionine-beta-synthase a (cbsa), mRNA | NM_001111232.1 | cbsaFor | TGCCAAAGAGCTGAAGGAAGGACA |
|  |  |  | cbsaRev | TCCACCACCAGGGTTTATTCACCA |
| hspa8 | Danio rerio heat shock protein 8 (hspa8), mRNA and HSP70-like | NM_001110403 | hspa8F | GATCGGCAGGAGGTTCGA |
|  |  |  | hspa8R | TTCCACTGCAACTTTTGGCTTT |
| mitfa | Danio rerio microphthalmia-associated transcription factor a (mitfa), mRNA | NM_130923.1 | mitfaF | ATTCTTGGGTTCATGGATGCA |
|  |  |  | mitfaR | CAGCTGGAGGAAGAGCATGAT |
| pmch | Danio rerio pro-melanin concentrating hormone (pmch) mRNA, partial cds | FJ392644.1 | pmchF | TGGATGAGCAACGTAACGTAGAA |
|  |  |  | pmchR | TGCCAGCAGGGCCTGTATAC |
| pomca | Danio rerio proopiomelanocortin a (pomca), mRNA | NM_181438.3 | pomcaF | GAAGAGGAATCCGCCGAAA |
|  |  |  | pomcaR | CCAGTGGGTTTAAAGGCATCTC |
| tyr | Danio rerio tyrosinase (tyr), mRNA | NM_131013.1 | TyrF | GGTGCCCTTCATCCCTCTCT |
|  |  |  | TyrR | AAACCGCTGACCTGGATCCT |
